# Biomechanical evaluation of novel construct for stabilizing subcapital femoral neck fractures: a synthetic bone model

**DOI:** 10.1186/s12891-025-08813-7

**Published:** 2025-07-04

**Authors:** Anjan R. Shah, Miguel A. Diaz, Douglas L. Cerynik, Blake Schultz

**Affiliations:** 1 Orthopaedic Trauma Service, Florida Orthopaedic Institute, 13020 N. Telecom Parkway, Tampa, FL USA; 2 Foundation for Orthopaedic Research & Education, 4115 W Spruce St. Suite 201, Tampa, FL USA; 3 Stabiliz Orthopaedics, 600 Eagleview Blvd, Exton, PA USA; 4 Texas Orthopedics Sports & Rehabilitation Associates, 911 W 38th St. Suite 300, Austin, TX USA

**Keywords:** Femoral neck fracture, Fracture fixation, Internal fixation, Neck shortening, Femoral head

## Abstract

**Background:**

High complication rates continue to be reported for femoral neck fractures, where moderate to severe shortening has been associated with pain and inferior functional outcomes. The objective was to evaluate the biomechanical performance of a novel femoral neck fracture fixation device by comparing to a clinically relevant construct using commercially available cannulated screws.

**Methods:**

Twelve Sawbones femurs were equally divided into two groups where Group I was instrumented with a new fixation system consisting of two partially threaded cannulated screws and a cross-screw and Group II was instrumented with three partially threaded cannulated screws. The fracture pattern resembled a Garden Type II fracture with superior fracture comminution with a 3° wedge. Mechanical testing was set to simulate heel strike. All constructs were preconditioned under a series of non-destructive tests (torsional, bending and compression). Thereafter, a step-wise increasing cyclic loading protocol to a maximum of 30,000 cycles or until failure. Fracture gap area collapse, rotation of the femoral head, screw displacement, failure modes and number of cycles to failure were compared between groups.

**Results:**

At 10,000 cycles, Group I had significantly larger fracture gap area when compared to Group II (*p* = 0.020). The fracture gap was also assessed after the additional fatigue protocol, and Group I continued to demonstrate enhanced ability to resist femoral neck shortening compared to Group II (*p* = 0.001). For femoral head rotation, Group I constructs demonstrated an average of 0.19° less rotation of the femoral head about the neck after 10,000 loading cycles (*p* = 0.88). From the zero position (0 N) to pre-load (50 N), Group I screws demonstrated less backout when compared to Group II. While this initial decrease in backout was not statistically significant (*p* = 0.47), with each subsequent loading step, Group I screws demonstrated significantly less displacement than Group II (*p* < 0.05). During the fatigue testing up to 30,000 cycles, samples in Group I failed at 20,286 cycles and Group II failed at 14,001 on average, however, this was not statistically significant (*p* = 0.19).

**Conclusion:**

Constructs in Group I outperformed Group II by demonstrating the ability to retain an increased fracture gap area during cyclical testing and withstand screw displacement.

**Level of evidence:**

Basic Science.

## Introduction

Internal fixation (IF) for minimally and non-displaced intracapsular femoral neck fractures remains a controversial topic as unpredictable outcomes persist. In particular, high complication rates continue to be reported with Garden I and II, subcapital fractures, where current fixation strategies lack axial and rotational stability leading to malunions and reoperations [1–5].

Studies such as the multi-center, randomized controlled FAITH trial demonstrated that over 30% of femoral neck fractures resulted in moderate to severe shortening [3, 6]. Neck shortening greater than 5 mm is associated with pain and inferior functional outcomes [3, 6–8]. Complications from rotation of the femoral head have been described using either three cannulated screws (CS) in an inverted triangle (IT) configuration and fixed angle devices, such as, sliding hip screws (DHS) [9–11]. Avascular necrosis (AVN) has been reported in 5–30% of cases where non-displaced neck fractures were repaired using IF [4, 12, 13] Additionally, symptomatic hardware prominence continues to be a reported complication [2, 6].

With reoperation rates ranging from 8 to 23%, many surgeons elect to proceed directly to arthroplasty for older patients [4, 14, 15]. For elderly patients with chronic comorbidities, newer IF devices have been shown to be advantageous to hemiarthroplasty [16]. For middle-age patients, IF would be valuable if it could reliably restore function, while limiting postoperative complications and postponing the need for arthroplasty [17].

There continues to be a need to improve mechanical stability while mitigating the challenges often associated with current fixation methods. Therefore, the purpose of this study was to biomechanically investigate whether a new device can provide enhanced stability, controlling femoral neck shortening, while resisting femoral head rotation and screw displacement compared to the common three-screw IT. It was hypothesized that the new device would minimize femoral neck shortening (measured by fracture gap), control femoral head rotation, experience less screw displacement and improve failure mechanics.

## Materials and methods

### Study specimen

**Fig. 1:**
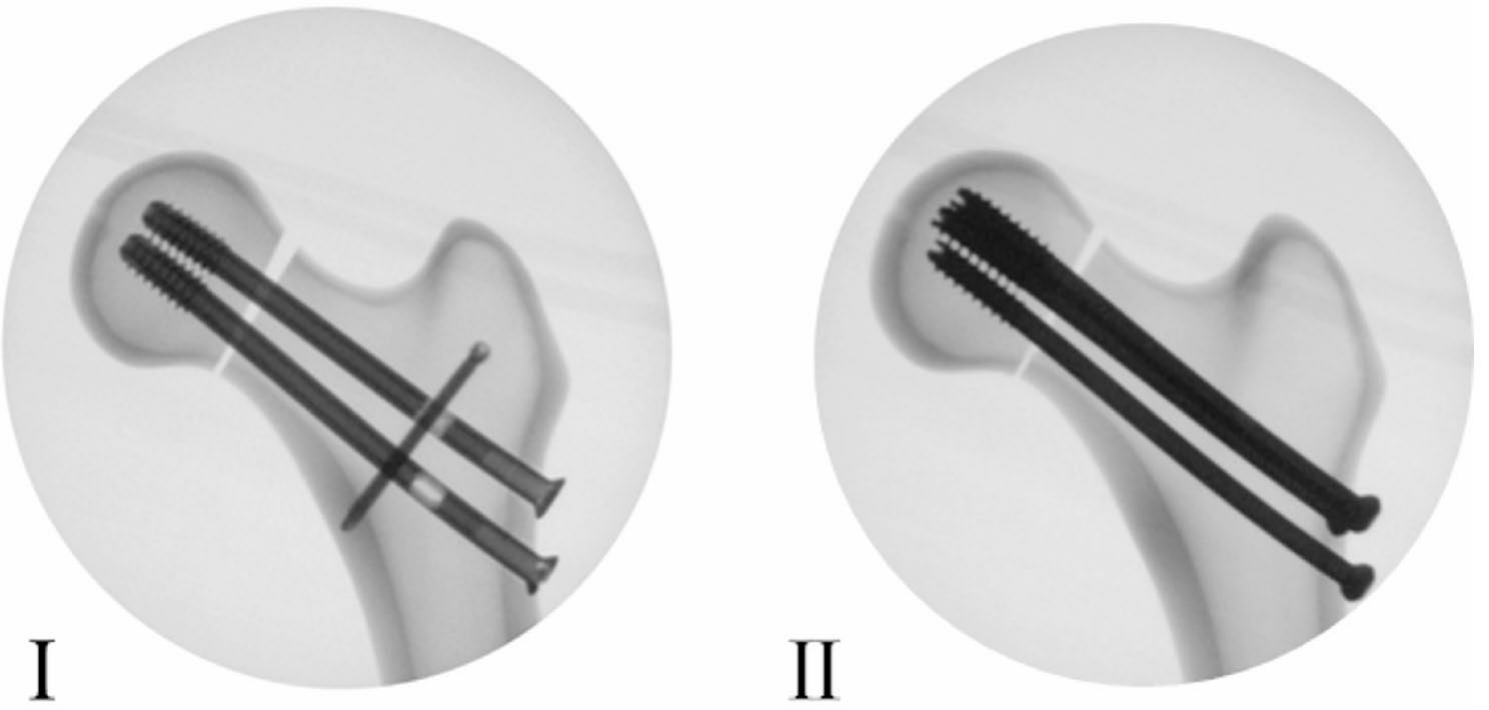
Radiographic example of Sawbones instrumented with (I) the SimpliFix (SF) construct and (II) the inverted triangle construct

A total of 12 Sawbones femurs (Model #3403, medium-Left; Pacific Research Laboratories, Vashon, WA) were divided into two repair groups. The femurs included were 4th generation composite bone models consisting of short fiber filled epoxy simulating cortical bone (Density: 102 PCF; Compressive Strength: 157 MPa; Compression Modulus: 17 MPa) with a 17 PCF density cancellous foam core and 13 mm canal. For Group I, the investigational constructs (SF, *n* = 6) were instrumented using two ø8.3 mm partially threaded cannulated screws with a slotted hole and one ø3.7 mm cross screw (SimpliFix; StabilizOrtho, Exton PA). For Group II, the control constructs (CS, *n* = 6) were instrumented using three ø6.5 mm partially threaded cannulated screws (Smith + Nephew, Memphis TN) in a clinically relevant triangle fashion (Fig. 1).

## Model Preparation and instrumentation

**Fig. 2:**
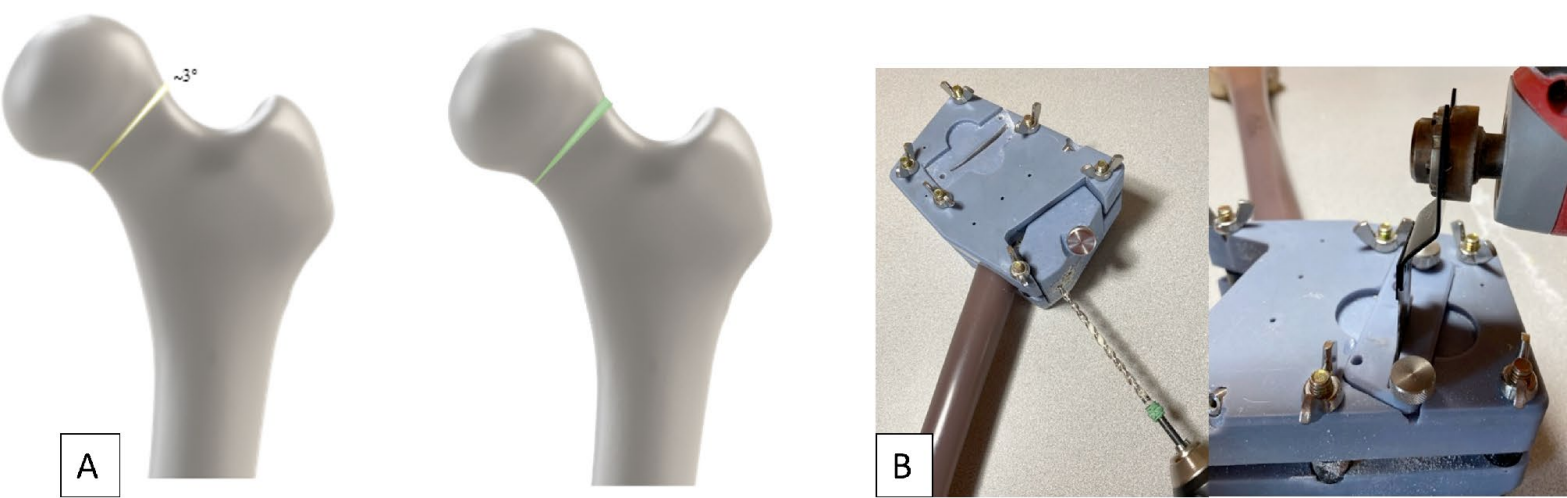
(**A**) Illustration of fracture creation to create a 3° wedge where the fracture gap area (in green) is indicative of femoral neck shortening. (**B**) Custom jig used for drilling and cutting

For reproducibility, all constructs were prepared using a customized jig that allowed standardized clamping sites to the femur. The fracture pattern developed was modeled from a Garden Type II fracture with superior fracture comminution. Using the customized jig, a blade of 1.15 mm thickness was used to cut along the cutting guide to create the 3-degree wedge (Fig. 2). This represents a worst-case scenario for Garden Type II fractures. After fracture creation, to standardize placement, pilot holes were predrilled (Cannulated screws were drilled using ø6.2 mm drill and cross screw used a ø2.7 mm drill.) by passing the drill bit through drill guide to the depth pre-determined via CAD models to. Each sample was then stabilized using the respective instrumentation guides with SimpliFix constructs having the cross screw placed at the distal end of the slotted hole.

## Specimen potting

After instrumentation, each sample was shortened to 18 cm with an oscillating saw and potted distally using high strength resin (Bondo body filler; 3 M Collision Repair Solutions, St. Paul, MN) in a positioning ball joint. The ball joint allows the sample to be locked in the sagittal plane in 10 degrees of flexion and 10 degrees of adduction to mimic heel strike [18]. Proximally, the femoral head was directly loaded using an acetabular component of a metal-on-metal hip prosthesis.

## Biomechanical test procedure

### Preconditioning loading

**Fig. 3:**
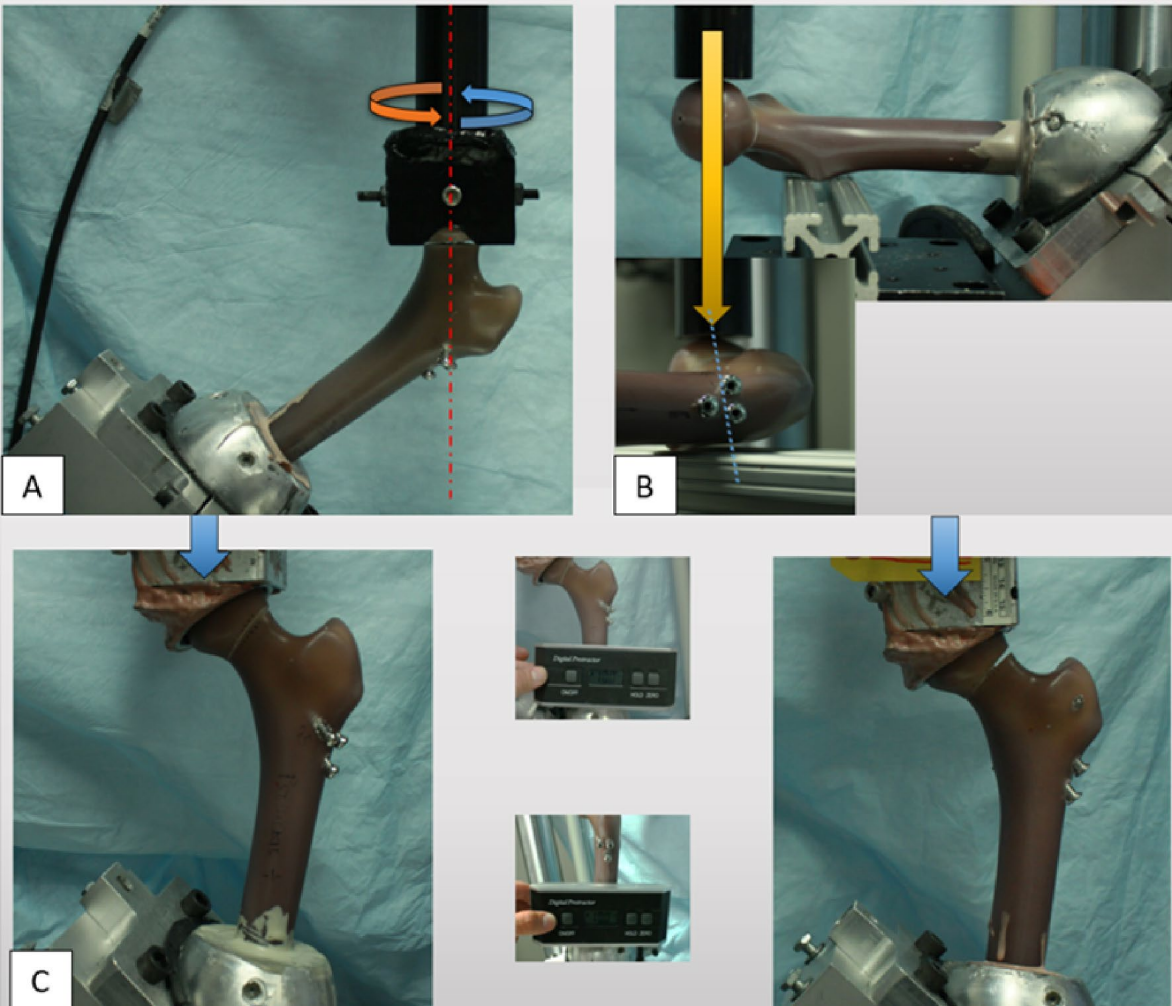
Example of instrumented specimen mounted in test frame where (**A**) preconditioning torsion, (**B**) preconditioning bending and (**C**) preconditioning and dynamic axial compression test

Based on previous studies [19], a series of non-destructive, preconditioning tests (torsional, bending and compression) were performed prior to dynamic testing on all specimen (Fig. 3). Load was applied to the femoral head with an actuator and a 5-kN load cell of a servohydraulic test frame (MTS Bionix; MTS Inc., Eden Prairie, MN). For torsional preconditioning, a torque at a rate of 1°/s was applied to femoral head until +/- 5° rotation around the longitudinal axis of the femoral neck [from neutral position, (+) 5° and (-) 5°] (Fig. 3A). Cantilever bending (anteroposterior loading) was performed with the samples placed horizontally with a support beam under the minor trochanter and load applied anteriorly (Fig. 3B). Lastly, for axial compression, samples were mounted vertically in 10° adduction and 10° flexion (Fig. 3C). All preconditioning tests were performed with a minimum axial preload of 30 N and repeated three times. For bending and compression testing, the maximum axial load was 500 N at a rate of 200 N/s [19].

### Dynamic testing

**Fig. 4:**
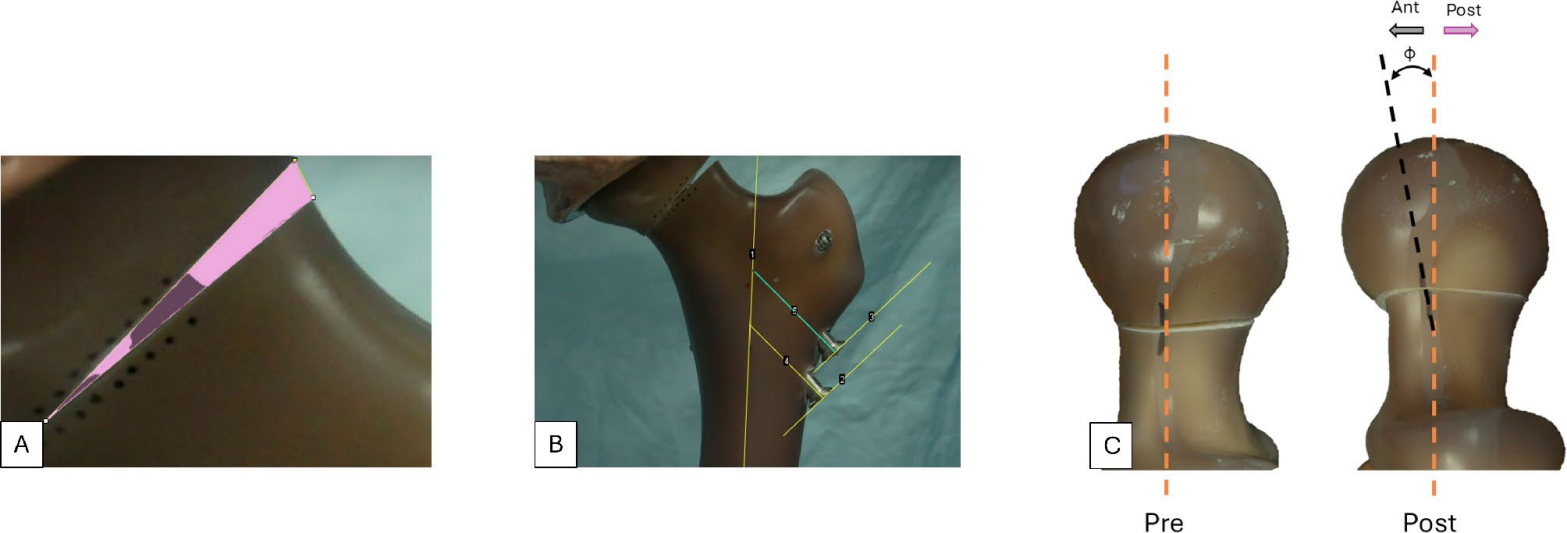
(**A**) Illustrates the shaded region representing the fracture gap area; (**B**) is an example of screw displacement (backout) measurement where the center of femur IM canal is used as reference; and (**C**) is an example of the head rotation calculation where the central axis is reference (in orange). All measurements were performed pre and post loading

Following preconditioning loading, while still oriented for axial compression, vertical and sinusoidal loading was applied through the acetabular component in a similar fashion to other authors [20–24]. Cyclic testing was performed under increasing axial load until failure [22, 25] Baseline loading was performed for 10 cycles between 50 and 100 N at a frequency of 0.5 Hz. Thereafter, a step-wise increasing cyclic loading protocol based on reported body weight (BW) for the Sawbones model was followed. Each load step was at frequency of 1 Hz in increments of 1,000 cycles until 85% BW (658 N) was achieved, whereafter each construct ran 4,000 cycles at 85% BW. Next, constructs continued in a step-wise loading fashion until 2X BW (1,646 N), whereafter each construct ran 3,124 cycles at 2X BW. After the completion of 10,000 cycles, an additional fatigue protocol was performed that continuously loaded the constructs at 2X BW to a maximum of 30,000 cycles or until failure. Any construct completing the 30,000 cycles was then ramped to failure at a rate of 1 mm/s. Construct failure was defined by actuator displacement > 15 mm (from baseline displacement) or by catastrophic failure. Catastrophic failure can be categorized as formation of fracture lines, catastrophic implant failure, or catastrophic specimen failure. Failure mode and cycles to failure were recorded. Stiffness data was collected from MTS load cell and linear variable differential transformer (LVDT), defined as the linear portion of the force-displacement curve. A high-definition camera (camera info) at stationary position was utilized to record video and image data at every stiffness level recording. Tracking markers were placed along the defect and femoral head to measure changes in rotation, and fracture gap area. Additionally, radiographs were taken of every sample before loading and after loading, where a custom mold was used to standardized sample orientation. These measurements were performed by using Image J for each loading condition (Fig. 4). Based on the anticipated failures from previous pilot samples, data for fracture gap area, femoral head rotation and screw backout was collected up to 13,000 cycles during the fatigue protocol.

### Statistical analysis

Subsequent to dynamic testing, fracture gap area, head rotation, screw displacement and number of cycles to failure were compared between groups. Averages expressed as arithmetic means and variation as standard deviations. To determine sample equivalence, all comparisons were evaluated using the F-test (alpha = 0.05) before applying the appropriate paired T-test with level of significance set to *p* < 0.05. The Pearson Correlation Coefficient was used to determine the relationship between outcomes, where appropriate.

## Results

### Preconditioning

No significant differences were found during the non-destructive, preconditioning tests between the SF and CS constructs for compression or torsional stiffness (*p* = 0.2297 and *p* = 0.5865, respectively). The CS construct was found to have higher bending stiffness compared to the SF construct (*p* < 0.001).

### Fracture gap area

After preconditioning and prior to dynamic loading SF constructs had 0.142 cm^2^ more fracture gap area when compared to CS constructs (*p* = 0.16).

**Fig. 5:**
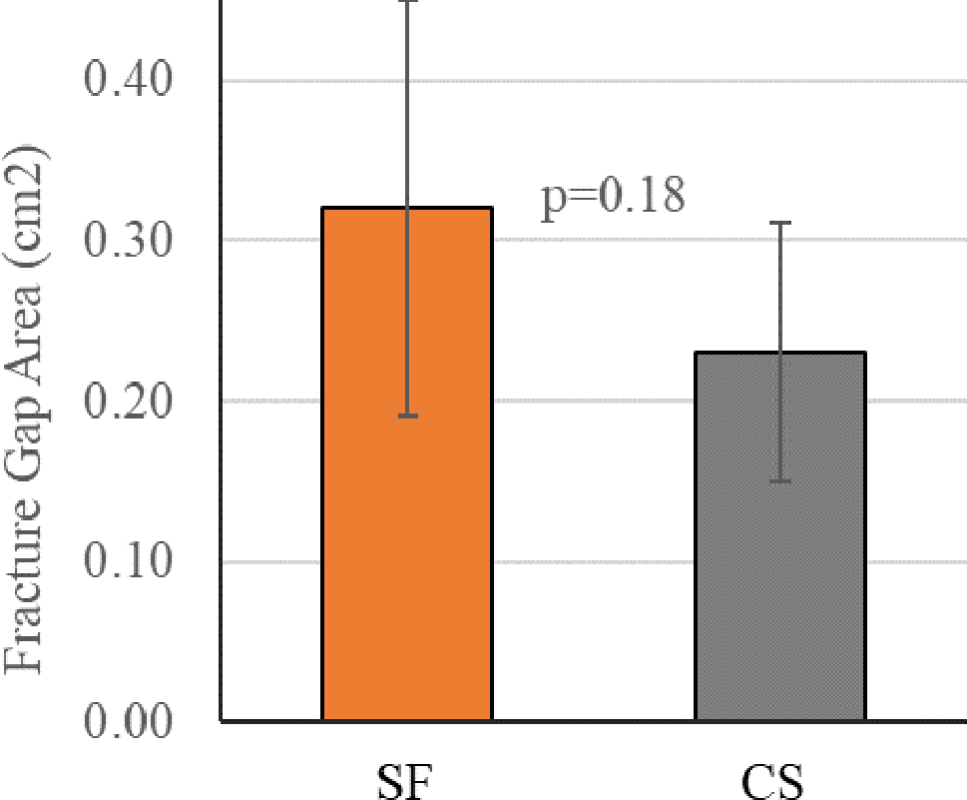
Comparison of fracture gap area at pre-load representing similar femoral neck lengths in both SF and CS constructs

Under minimum axial pre-load (50 N), SF had 0.090 cm^2^ larger fracture gap area when compared to predicate constructs (*p* = 0.18) (Fig. 5). The statistically similar fracture gap area seen in SF and CS constructs provides an equivalent baseline to assess the ability of SF to resist femoral neck shortening under repeat loading.

**Fig. 6:**
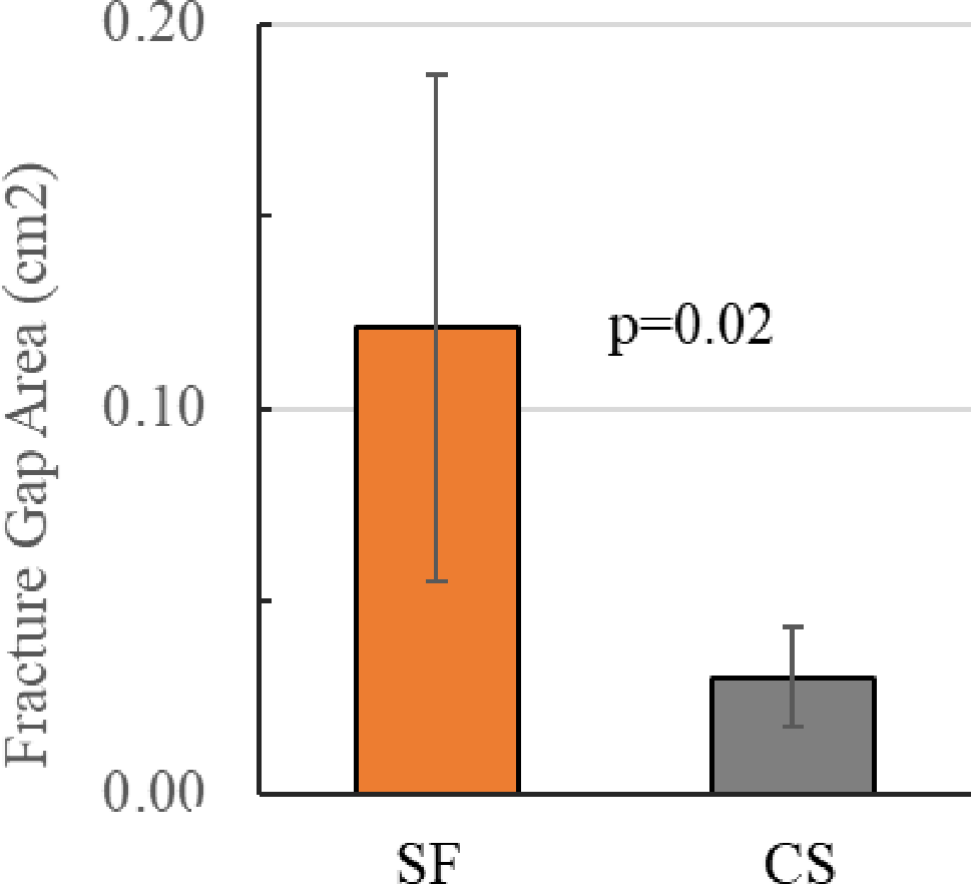
Comparison of fracture gap area after 10,000 loading cycles with SF preserving greater neck length than CS constructs

At the conclusion of 10,000 cycles (with 3,124 cycles at 2x BW, 1646 N), SF demonstrated an average fracture gap area that was 0.0908 cm^2^ greater than CS (*p* = 0.02) (Fig. 6).

**Fig. 7:**
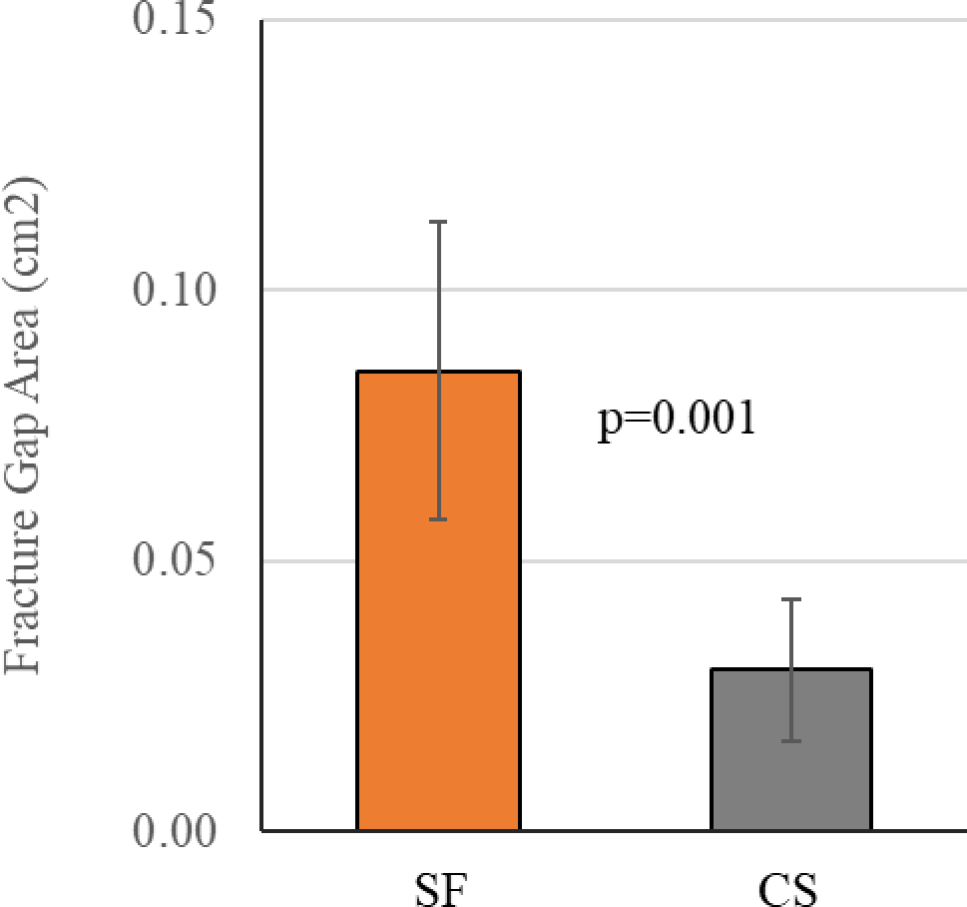
Comparison of fracture gap area after 13,000 loading cycles. SF maintains significantly more neck length than constructs with three CS

During the additional fatigue protocol, fracture gap area was assessed after 13,000 total cycles (with 6,124 cycles at 2X BW, 1646 N). All SF constructs survived to this point. Two CS constructs failed prior to 13,000 cycles. SF constructs demonstrated an average fracture gap area that was 0.0552 cm^2^ greater than CS constructs (*p* = 0.001) (Fig. 7).

### Screw displacement (Backout)

**Fig. 8:**
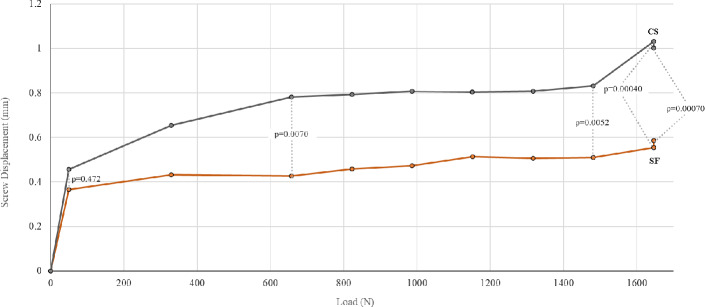
Graphic representation of screw displacement. SF (orange) had significantly less screw displacement under load compared to CS (gray)

**Fig. 9:**
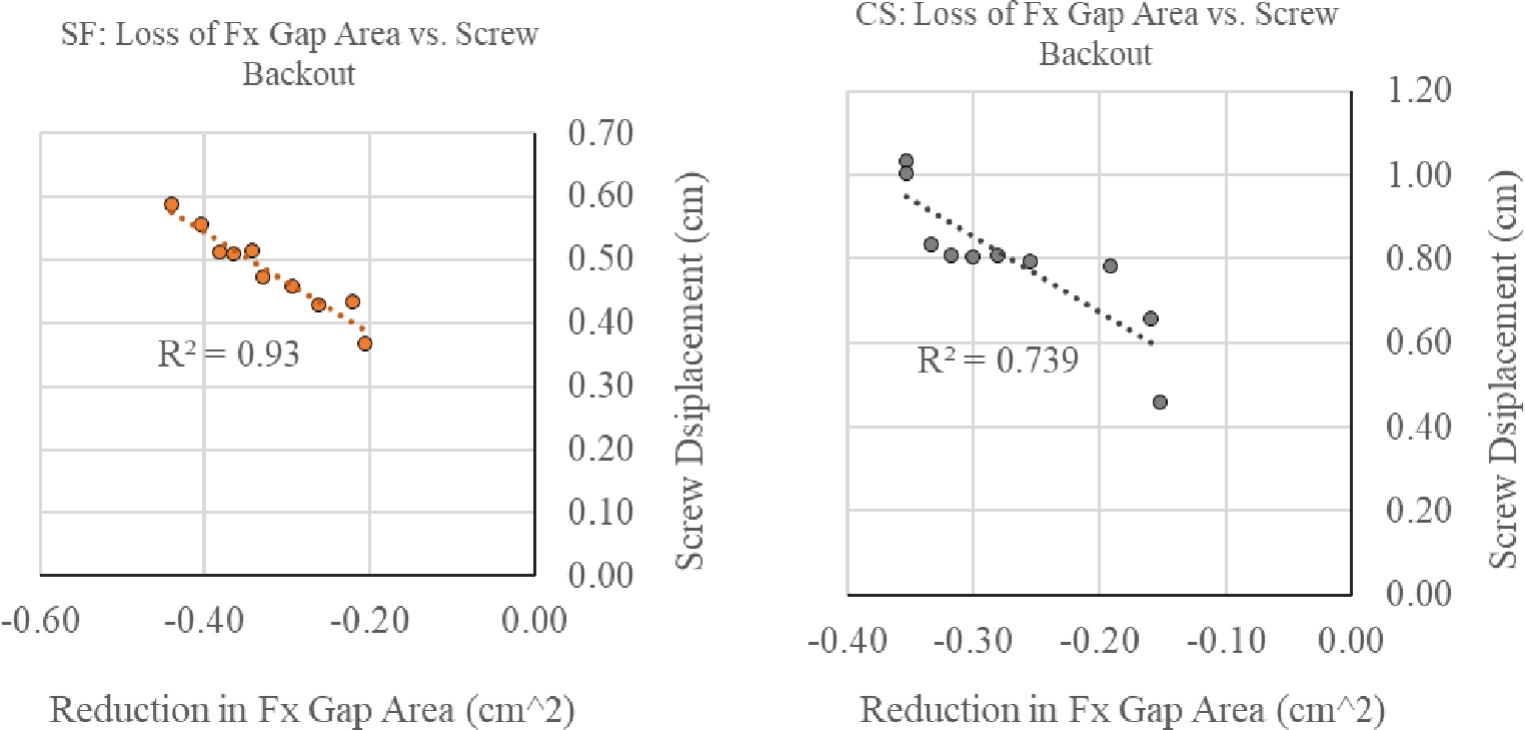
Pearson correlation coefficient scatter plots showing the relationship between fracture gap area (i.e., maintaining femoral neck length) and the screw displacement. The strong relationship indicates the importance of maintaining fracture gap area and corresponding screw displacement

From the zero position (0 N) to pre-load (50 N), SF demonstrated 0.0911 mm less backout when compared to CS (*p* = 0.47). With each subsequent loading step, SF demonstrated significantly less displacement (*p* < 0.05) than CS (Fig. 8). Reduction in fracture gap area demonstrated a strong negative correlation with screw displacement for both SF and CS constructs (*R*= -0.96 and *R*= -0.86, respectively; Fig. 9).

### Femoral head rotation

**Fig. 10:**
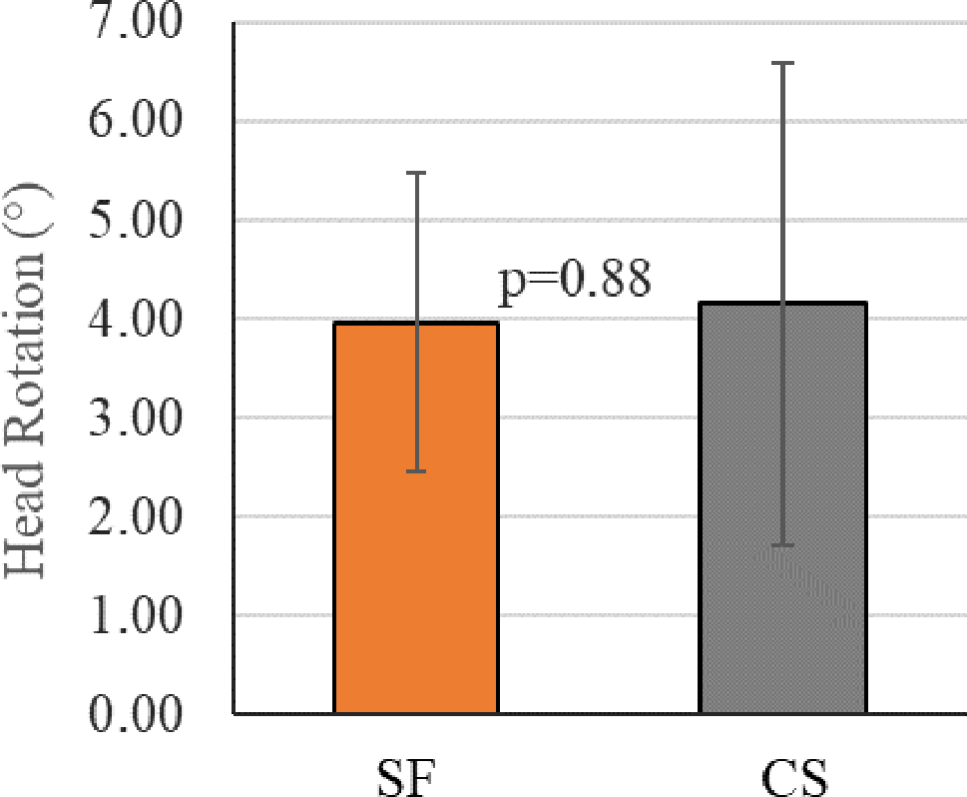
Graph demonstrating average change in femoral head rotation. SF demonstrated less femoral head rotation

The SF constructs demonstrated an average of 0.19° less rotation of the femoral head about the neck after 10,000 loading cycles (SF: 3.96 ± 1.51°, CS: 4.15 ± 2.43°; Fig. 10) (*p* = 0.88).

### Ultimate failure

SF constructs outperformed CS by 6,286 cycles, on average (*p* = 0.19). (Table 1) The CS failed on average around 14,001 cycles, with the earliest constructs failing after 12,900 and 12,914 cycles. The closest Fracture Gap Area reading of all other specimen occurred after 13,000 cycles. (Table 2)

**Fig. 11:**
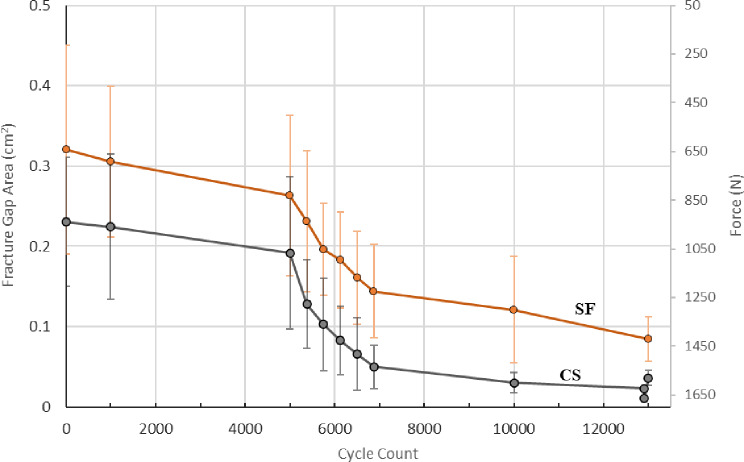
Comparison of average fracture gap area versus loading cycles where SF (in orange) resisted femoral neck shortening (i.e., SF had a larger fracture gap area) versus constructs containing three CS (in gray). SF ability to resist femoral neck shortening outperformed CS in terms of fracture gap area, load cycles, and load

During additional fatigue cycle testing (10,000 + cycles), all constructs failed except for one SF construct which was then ramped to failure and failed at 3,421 N. The amount of final head rotation could not be measured due to the failure mode, however the amount of fracture collapse was captured. At 13,000 cycles, SF constructs maintain a statistically significant average fracture gap area that is 0.0522 cm^2^ greater than that seen in CS constructs (*p* < 0.04; Fig. 11).

**Fig. 12:**
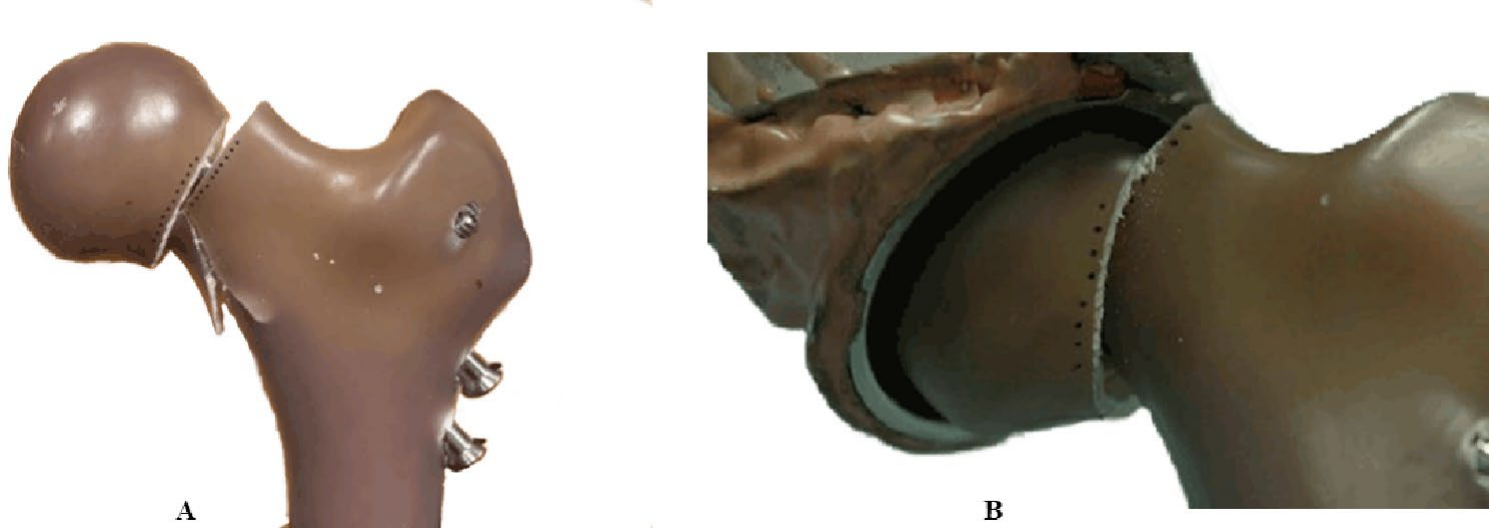
Illustration of failure modes where (**A**) a fracture of the Sawbone occurred in SF construct after repeat loading at 2X BW, and (**B**) rotational failure and femoral neck collapse of CS after repeat loading at 2X BW

SF constructs demonstrated enhanced survival under repeat loading compared to CS, with one SF construct surviving until catastrophic load to failure (40,000 cycles). No implants broke during fatigue or load to failure. The failure mode for the samples were predominantly fracture of the Sawbones at the inferior calcar. Additionally, some samples had excessive head rotation and loss of fracture gap area (i.e., femoral neck shortening) where the inferior surface of the neck was embedded into the femoral head. (Fig. 12) There was no implant breakage observed in any specimen.

## Discussion

This study compared two femoral neck fracture fixation systems using a clinically relevant biomechanical model simulating non-displaced Garden II fractures. The purpose was to investigate failure modes known to result in significant morbidity and reoperation, namely, femoral neck shortening, femoral head rotation and screw displacement. SF demonstrated the ability to control the fracture gap area throughout the loading protocol compared to the CS, supporting our hypothesis that the new device would minimize femoral neck shortening. Similarly, the SF had significantly less screw displacement with each subsequent loading step compared to the CS supporting our hypothesis. SF constructs outperformed CS by running out to higher cycle counts, while several CS samples exhibited the femoral neck cutting into the cancellous material of the femoral head at failure.

The randomized controlled FAITH trial reported a reoperation rate of 20–22% after IF of low energy femoral neck fractures (Garden I-IV) with either two, three, or four screws or DHS constructs. The reoperation rate for nondisplaced (Garden I & II) fractures was 16%, regardless of fixation type [4]. Dolatowski et al. [26] reported a complication rate of 24.3% and reoperation rate of 24% with two 8.0 mm cannulated screws. Lu et al. [27] reported a complication rate of 39% and reoperation rate of 22% with three 6.5 mm CS. More recently, a retrospective study by Gaski et al. reported a 15% reoperation rate [5].

The FAITH trial demonstrated no difference in the rate of reoperation or complications between cannulated screws and dynamic hip screws (DHS), furthering the debate over technique and implant choice [4, 16, 17, 28–31] Schottell et al. [31] used FAITH trial data to show that no difference in revision rates existed between two, three, or four screws, but significantly lower revision rates were seen with a three-screw IT compared to three-screw triangle formation. While achieving actual cortical abutment of three diverging screws is technically demanding, Schottell’s findings correspond with biomechanical studies reporting enhanced stability using this technique [32]. Conversely, studies comparing two versus three cannulated screws demonstrate similar findings, questioning superiority of the IT technique [1, 31, 33, 34]. More recently, the IT technique has been scrutinized for cortical breach of the posterosuperior screw in the area of the lateral epiphyseal artery, the so called *in-out-in* screw, adding concern to risk of AVN [35, 36]. Additionally, Gaski et al. [5] reported a higher risk of reoperation with IT constructs consisting of all partially threaded screws (17.0%) compared to using at least one fully threaded screw (7.5%), inferring that better stability trends towards less reoperations.

While it is accepted that nondisplaced femoral neck fractures heal with compression, the amount needed has not been quantitated. As current techniques do not control neck shortening, malunions after fixation are well reported due to uncontrolled neck shortening. Cronin et al. [2] reported that 42% of Garden I and 63% of Garden II fractures demonstrated greater than 10 mm of fracture collapse when treated with in situ CS. Felton et al. [3] reported results from the FAITH Trial, citing 24% of Garden I and II fractures healed with moderate (> 5–10 mm) to severe (> 10 mm) shortening, with declining outcomes associated with increased neck shortening. These findings are similar to numerous other reports citing inferior hip function and decreased patient satisfaction resulting from uncontrolled neck shortening and hardware prominence [6–8, 37]. More recent implants, such as the Femoral Neck System (FNS), as reported by Cintean et al. [16], continue to show that neck shortening > 5 mm (19%) remains an unsolved issue.

In the current study, femoral neck shortening was quantified by assessing fracture gap area under clinically relevant loading. The presence of the cross screw in SF constructs likely resulted in greater resistance to femoral neck shortening compared to traditional CS. SF demonstrated significantly less screw displacement (i.e., backout) compared to CS, correlating with its ability to maintain fracture gap area (i.e., femoral neck length). It is of clinical relevance to understand the fracture gap area remaining across each loading steps as indicative of a construct’s ability to resist femoral neck shortening, especially under repetitive loading. Clinically, the enhanced ability to control femoral neck shortening and screw backout may reduce complications related to malunion, hardware prominence, postoperative pain and gait disturbance.

Failures related to varus deformity, rotation of the femoral head and screw cutout continue to be reported [10, 19, 38]. Femoral head rotation and/or postoperative varus deviation represents an unstable construct and, probable, impending failure [9]. Cutout of legacy CS and DHS implants is well documented [39, 40], while newer FNS implants have seen a cutout rate between 4.9 and 13.8% as reported by Davidson et al. [41] and Cintean et al. [16]. In the current study, SF had less femoral head rotation than CS although this was not statistically significant. Enhancing the ability of constructs to maintain rotational stability under load may help preserve fracture reduction and alignment in the clinical setting.

Additionally, AVN is a common complication for non-displaced femoral neck fractures being reported in up to 29% of cases [4, 42]. While bone quality and comorbidities are contributing factors, it may also be argued that implant choice, significant neck collapse, head rotation, and postoperative varus tilt have a role in fostering AVN [43], while cutout may be the result of bone necrosis [40]. In particular, AVN has been correlated with the use of large implants, like DHS, which may disrupt blood supply to the femoral head [4, 43]. Regardless of failure mechanism, revision often requires are major procedure such as total hip arthroplasty or hemiarthroplasty [14, 40, 44]. Implants that maintain femoral neck length while resisting head rotation may reduce the risk of revision surgery secondary to femoral neck shortening, gait disturbance and avascular necrosis of the femoral head.

As arthroplasty has been a commonly accepted treatment option for displaced (Garden III and IV) femoral neck fractures, some have advocated broader indications for it, versus IF, in nondisplaced femoral neck fractures [45, 46]. These recommendations cite lower reoperation rates due to no loss of fixation, AVN or symptomatic screw backout. Interestingly, these same studies report no statistically significant difference between the two modalities at 12-months in regards to Harris Hip Scores and patient reported outcomes. Slobogean et al. [7] supports this hypothesis with lower reoperation rates in arthroplasty versus CS (5.8 versus 13.3%, respectively), but functional outcomes were not statistically significant different, the need for blood transfusion was almost 10x greater in the arthroplasty group (48.6 versus 4.8%), mortality rate was not significantly increased at 1-year for arthroplasty and they were unable to determine whether arthroplasty had an increased risk of earlier perioperative mortality. It should be noted that both studies included randomized control trials by Lu et al. and Dolatowski et al., who each exclude patients with ASA IV designations [26, 27]. Lu et al. [27] states that their findings of no difference between the two groups in terms of mortality and survival time maybe due to removing patients with poor surgical tolerance, thus contributing to an increased survivability. Cintean et al. [16] reported on IF using FNS versus hemiarthroplasty for Garden I fractures with ASA score 3 and higher, demonstrating additional blood transfusions and significantly longer hospitalization in the hemiarthroplasty group. Arthroplasty and hemiarthroplasty, are not without their own associated risks. Further study is warranted to determine indications for their use in nondisplaced femoral neck fractures.

While the use of composite bone surrogates for precisely comparing implants is well accepted [19], confirmation of outcomes using osteoporotic cadaver femurs may be warranted. The wedge osteotomy created in the superior femoral neck represented the comminution seen clinically with Garden II fractures. Although creating a worse-case construct, this missing wedge may have initially increased construct instability. Further assessment of undisplaced fractures using a stable fracture pattern may be warranted.

While the load set-up used has been routinely reported in the literature, it represents one particular profile that may not be fully replicable clinical scenarios in terms of loading cycles (steps), magnitude and direction of force. The study protocol loaded up to 2xBW (1,646 N) and can be considered worst case scenario as comminuted fracture healing cannot be modeled. This loading model was chosen to represent single leg stance through walking phase where the number of cycles approximates the number of steps taken over a 4-week to 6-week time period [47–49].

Internal fixation of nondisplaced (Garden I and II) hip fractures can have better outcomes. Current fixation strategies lack axial and rotational stability leading to fixation failures, malunions and reoperations. Even in cases of healed fractures, there are clinical consequence of excessive neck shortening. Can we do better without rushing to arthroplasty?

Limiting neck shortening, lowering reoperation rates, improving outcomes by enhancing stability while maintaining the perioperative risk benefits of the current “percutaneous” technique should be the goal of innovative implants. SF provides a simple technique and similar limited perioperative risks, adds axial stability, trends toward greater rotational stability, avoids the demands and complications of a third screw and may potentially lower AVN rates. Clinically, this may result in less reoperations, better outcomes, and the avoidance of arthroplasty.

**Table 1:** Total number cyclic loading cycles per specimen

Specimen No.	SF	CS
**1**	18,865	13,389
**2**	13,168	14,242
**3**	14,870	12,900
**4**	40,000	13,623
**5**	13,682	12,914
**6**	21,136	16,939
**AVG (SD)**	20,286.8 (± 10,149)	14,001.2 (± 10,149)

**Table 2:** Comparison of fracture gap area at nearest timepoint to failure of first predicate

Specimen No.	SimpliFix^A^	CS
1	0.086	0.022^A^
2	0.05	0.04^A^
3	0.099	0.023^B^
4	0.13	0.043^A^
5	0.076	0.011^B^
6	0.069	0.04^A^
AVG	0.0850	0.0298

A. Fracture gap area measured at 13,000 cycles

B. Specimen failed prior to 13,000 cycles. Area represents last attained value

## Data Availability

The datasets used and/or analyzed during the current study are available from the corresponding author on reasonable request.
